# Single CD28 stimulation induces stable and polyclonal expansion of human regulatory T cells

**DOI:** 10.1038/srep43003

**Published:** 2017-02-22

**Authors:** Xuehui He, Ruben L. Smeets, Esther van Rijssen, Annemieke M. H. Boots, Irma Joosten, Hans J. P. M. Koenen

**Affiliations:** 1 Laboratory of Medical Immunology, Department of Laboratory Medicine, Radboud university medical center, Nijmegen, The Netherlands; 2 College of Computer Science, Qinghai Normal University, Xining, Qinghai, China; 3 Laboratory of Clinical Chemistry, Department of Laboratory Medicine, Radboud university medical center, Nijmegen, The Netherlands; 4 Department of Rheumatology and Clinical Immunology, University of Groningen, University Medical Center Groningen, Groningen, The Netherlands

## Abstract

CD4+FOXP3+ Treg are essential for immune tolerance. Phase-1 clinical trials of Treg-therapy to treat graft-versus-host-disease reported safety and potential therapeutic efficacy. Treg-based trials have started in organ-transplant patients. However, efficient *ex vivo* expansion of a stable Treg population remains a challenge and exploring novel ways for Treg expansion is a pre-requisite for successful immunotherapy. Based on the recent finding that CD28-signaling is crucial for survival and proliferation of mouse Treg, we studied single-CD28 stimulation of human Treg, without T cell receptor stimulation. Single-CD28 stimulation of human Treg in the presence of recombinant human IL-2(rhIL-2), as compared to CD3/CD28/rhIL-2 stimulation, led to higher expression levels of FOXP3. Although the single-CD28 expanded Treg population was equally suppressive to CD3/CD28 expanded Treg, pro-inflammatory cytokine (IL-17A/IFNγ) production was strongly inhibited, indicating that single-CD28 stimulation promotes Treg stability. As single-CD28 stimulation led to limited expansion rates, we examined a CD28-superagonist antibody and demonstrate a significant increased Treg expansion that was more efficient than standard anti-CD3/CD28-bead stimulation. CD28-superagonist stimulation drove both naïve and memory Treg proliferation. CD28-superagonist induction of stable Treg appeared both PI3K and mTOR dependent. Regarding efficient and stable expansion of Treg for adoptive Treg-based immunotherapy, application of CD28-superagonist stimulation is of interest.

Regulatory T cells are crucial for immune homeostasis and tolerance. These cells are typically characterized by expression of the transcription factor FOXP3, and have been shown to play an important role in the prevention of graft-versus-host-disease (GvHD), transplantation rejection and autoimmunity^1^. Treg-based immunotherapy applying *ex vivo* expanded naturally occurring Treg (nTreg) prevented pathology in a wide variety of mouse models^2^,^3^,^4^,^5^. The prospects of these studies supported phase-I clinical trials of Treg-based cell therapy in stem cell transplantation (SCT), which reported safety and potential therapeutic efficacy^6^,^7^,^8^. This success promoted the recent initiation of Treg-based immunotherapy in solid organ transplantation (The One Study, ThRIL). Notwithstanding the first successes in the translation of Treg therapy to the clinic, successful *ex vivo* expansion of a stable suppressive Treg population in sufficient numbers still remains one of the key challenges in clinical practice in order to achieve full clinical efficacy. Combined T cell receptor (TCR)/CD3 stimulation and CD28 in the presence of exogenously added recombinant human IL-2 (rhIL-2) is commonly used to expand human Treg^9^,^10^. This procedure can lead to high cell yields, but also revealed Treg plasticity, characterized by loss of FOXP3 and the ability of the Treg to convert into (pathogenic) pro-inflammatory cytokine (IL-17A and IFNγ) secreting cells^11^,^12^,^13^. This prompted the search for agents that promote Treg stability. High level expression of FOXP3 is important for optimal Treg function. This is maintained by hyper-demethylation of a noncoding CpG motif within the *FOXP3* gene upstream of exon-1 that is referred to as the Treg-specific demethylated region (TSDR)^14^. The mTOR inhibitor rapamycin is often added to expansion cultures to enhance FOXP3 expression and prevent outgrowth of contaminating conventional T cells^15^,^16^,^17^. However, although rapamycin works favorably on Treg function, addition of rapamycin generally leads to lower overall Treg cell yields^17^. Therefore, there is a need for novel approaches that yield high numbers as well as highly suppressive and stable Treg.

It is well appreciated that CD28 stimulation plays an important role in the development of FOXP3+ cells in the thymus^18^,^19^. Notably, recent data obtained in Treg-specific CD28 conditional knockout mice, indicates that CD28 signaling is also crucial for peripheral Treg survival, proliferation and suppressor function^20^. The intrinsic CD28 deficiency in peripheral Treg resulted in autoimmunity that could be prevented by supplementation with CD28-sufficient Treg^20^. In rodent models it was demonstrated that CD28 stimulation promotes expansion of CD4+CD25+ Treg^21^,^22^. Interestingly, artificial antigen-presenting cells modified to express the natural CD28 ligand CD86, as compared to anti-CD3/anti-CD28 bead stimulation induced superior proliferation of human cord blood derived Treg^23^. Recently, Tabares *et al*. showed that *in vitro* stimulation of human PBMC by low-dose CD28 superagonist (TGN1412) selectively activated Treg^24^. We hypothesize that CD28 signaling in the absence of CD3 stimulation might play an important role in human Treg homeostasis and that single-CD28 stimulation might drive stable expansion of human Treg, to be used for Treg-based immunotherapy. Here, we demonstrate that single-CD28 stimulation in the absence of TCR (CD3) stimulation, but in the presence of exogenously added rhIL-2 promotes superior FOXP3 expression and prevents the production of pro-inflammatory cytokines IL-17A and IFNγ. The use of CD28-superagonistic mAbs further promotes polyclonal Treg expansion, to even higher levels as observed in case of classical CD3/CD28 stimulation. The mechanism resulting in CD28-superagonist mediated Treg stability depends on differential PI3K and mTOR signaling, since selective PI3K-inhibition restores the cytokine producing potential while mTOR inhibition led to reduced FOXP3 expression levels.

## Results

### Single-CD28 stimulation of FACS-sorted CD4+CD25^high^ Treg induces proliferation and high level expression of FOXP3

**Figure 1:**
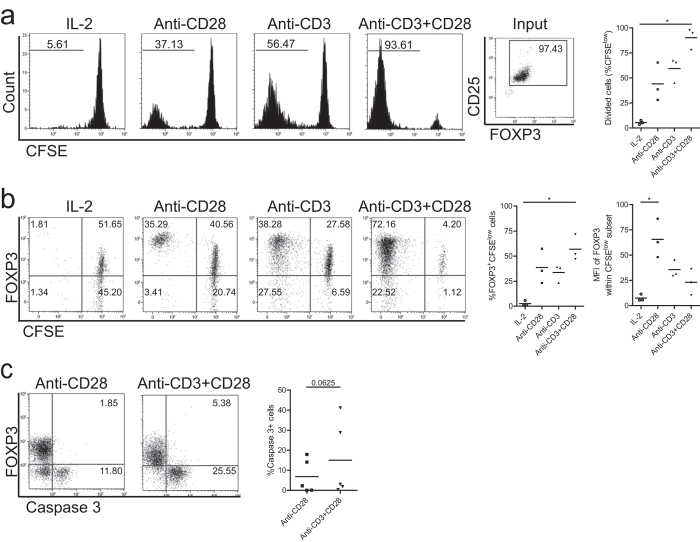
Single-CD28 stimulation induces Treg proliferation and promotes high level FOXP3 expression. Flow cytometry of FACS-sorted human Treg (CD4+CD25^high^) that were labeled with CFSE and stimulated with soluble CD28 mAb, plate bound CD3 mAb or both (Anti-CD3+CD28) in the presence of rhIL-2. As a control Treg cultured in the presence of rhIL-2 only were included. Cell division indicated by the dilution of CFSE (**a**), intracellular expression of FOXP3 (**b**) and active caspase 3 (**c**) were determined at day 7 of the cultures. Numbers within the histograms indicate the percentage of divided cells (**a**) and numbers within the quadrant show the percentage of positive cells (**b,c**). Cumulative data of Treg proliferation (**a**, right panel), the median fluorescence intensity (MFI) of FOXP3 (**b**, right panel), and the percentage of apoptotic cells (**c**) right panel) are also shown. Bar in cumulative data indicates the mean value. Kruskal-Wallis followed by Dunns post-hoc test (**a**) n = 3; b, n = 3) and Wilcoxon signed-rank test (**c**, n = 5) were used for statistical analysis. *P < 0.05.

To assess the ability of single-CD28 stimulation to stimulate human Treg proliferation, highly purified FACS-sorted human CD4+CD25^high^ Treg were labeled with CFSE and stimulated with soluble CD28 monoclonal antibody (mAb), plate-bound CD3 mAb or both in the presence of rhIL-2 for 7-days. Thereafter, the cell division of Treg as indicated by the dilution of CFSE was determined using flow cytometry. Single-CD28 stimulation in the absence of T cell receptor ligation was indeed capable to drive Treg proliferation (44.1% ± 19.2, mean ± SD, Fig. 1a). Combined plate-bound CD3 plus soluble CD28 mAb (anti-CD3+CD28) stimulation resulted in the highest level of proliferation. Intriguingly, the dividing Treg that were observed following CD28 stimulation showed higher FOXP3 expression levels at day 7 of culture (MFI, 65.6 ± 19.1) than the dividing Treg that were observed following anti-CD3 (MFI, 35.6 ± 8.5) or anti-CD3+CD28 (MFI, 23.1 ± 12.9, mean ± SD, Fig. 1b) stimulation. As it has recently been shown that CD28 signaling is required for post thymic survival and proliferation of mouse Treg^20^, we analyzed if single-CD28 stimulation in the absence of CD3 stimulation influenced Treg survival by measuring the intracellular expression of active caspase-3 as an indicator of Treg cell apoptosis. Indeed, single-CD28 stimulation in the presence of exogenously added rhIL-2 resulted in a slightly lower, albeit not significant, percentage of apoptotic cells as compared to anti-CD3+CD28 stimulated Treg (Fig. 1c).

### Single-CD28 stimulated Treg maintain suppressive capacity and TSDR demethylation

**Figure 2:**
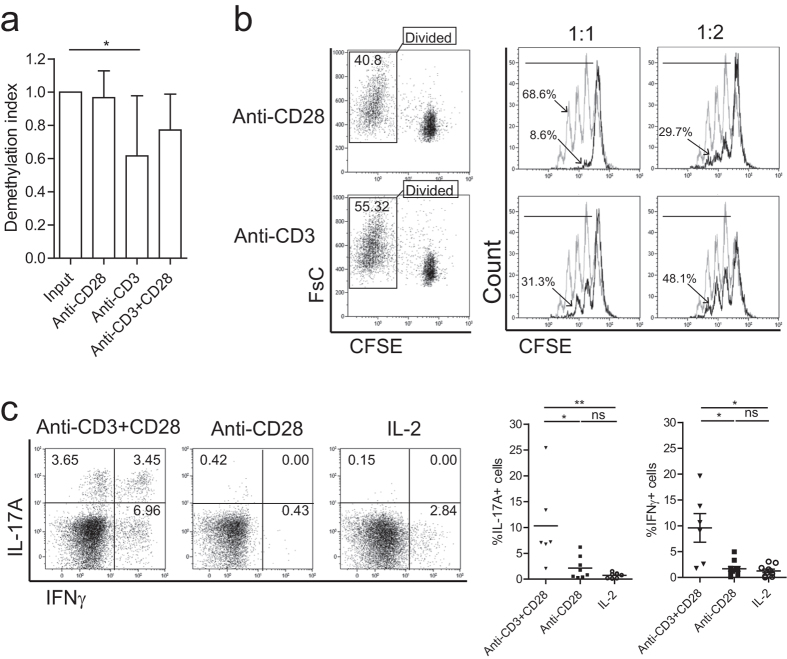
Single-CD28 stimulated Treg reveal a highly demethylated *FOXP3* gene and profound suppressor function. (**a**) FACS-sorted human Treg were stimulated with soluble CD28 mAb, plate bound CD3 mAb or both (anti-CD3+CD28) in the presence of rhIL-2 for 7-days. Thereafter, cells were harvested and the demethylation status of *FOXP3* gene was analyzed using bisulfate sequencing. n = 6–7. (**b**) CFSE-labeled FACS-sorted Treg were stimulated as described above. The divided cells (CFSE low population) were re-sorted (left panel) at day 7 of the cultures, and subsequently their suppressive function was analyzed in a co-culture suppression assay. Overlay histograms show the inhibition of responder T cells (Tresp) proliferation following the addition of graded doses of Treg. Numbers indicate the percentage of divided responder T cells. Grey line: stimulated Tresp, Black line: co-cultured with Treg of interest. The ratio of Treg:Tresp are indicated on the top. Representative experiment of n = 3 individual experiments conducted with cells obtained from different donors are shown. (**c**) Intracellular staining of cytokine IL-17A and IFNγ after additional stimulation with PMA, ionomycin, and Brefeldin-A for 4 hours. Numbers within the quadrant indicate the percentage of positive cells. Dotplots show a representative experiment of n = 6–10 individuals as shown in the cumulative data graph (right panel). Kruskal-Wallis followed by Dunns post-hoc test was used for statistical analysis (**a,c**). *P < 0.05; **P < 0.01; ns: not significant.

Thymus derived Treg contain a highly demethylated TSDR, which is crucial for maintaining a stable level of FOXP3 expression and suppressor capacity^14^. To explore if single-CD28 stimulation modifies the methylation status of the TSDR, bisulphite sequencing was applied. CD28-stimulated Treg revealed a tendency towards higher levels of TSDR hyper-demethylation (demethylation index 0.97 ± 0.16) as compared to CD3+CD28 (0.77 ± 0.22) or CD3-stimulated (0.62 ± 0.36, mean ± SD, Fig. 2a) Treg. To assess if these expanded Treg maintained their suppressive capacity, CFSE-labeled FACS-sorted Treg were stimulated using CD28 or CD3 (for comparison) in the presence of rhIL-2, and the dividing Treg population, as indicated by the CFSE-dilution, was FACS-sorted at day 7 and analyzed for their suppressor capacity using CFSE-based co-culture suppression assays. Both CD28- and CD3- expanded Treg preserved their capacity to suppress the proliferation of responder cells (Fig. 2b).

### Single-CD28 stimulated Treg have a reduced pro-inflammatory cytokine producing potential

In recent years, it has become clear that FOXP3+ Treg may show functional plasticity as indicated by their capacity to produce pro-inflammatory cytokines such as IL-17A and IFNγ under specific microenvironmental stimuli^11^,^12^,^25^. Thus, we analyzed the IL-17A and IFNγ producing potential of human FACS-sorted Treg that were stimulated with single-CD28 or anti-CD3+CD28 mAb. Single-CD28 stimulated Treg revealed lower percentages of IL-17A producing cells, as compared to anti-CD3+CD28 (2.2% ± 2, versus 10.3% ± 8 for CD3+CD28, mean ± SD), and these cells hardly produced IFNγ (1.7% ± 1.5, mean ± SD, Fig. 2c). This indicates that single-CD28 stimulation mediated expansion of human Treg results in a Treg population with increased stability as compared to anti-CD3+CD28 expanded Treg.

### CD28-superagonist stimulation promotes a stable Treg phenotype and leads to profound polyclonal Treg expansion

**Figure 3:**
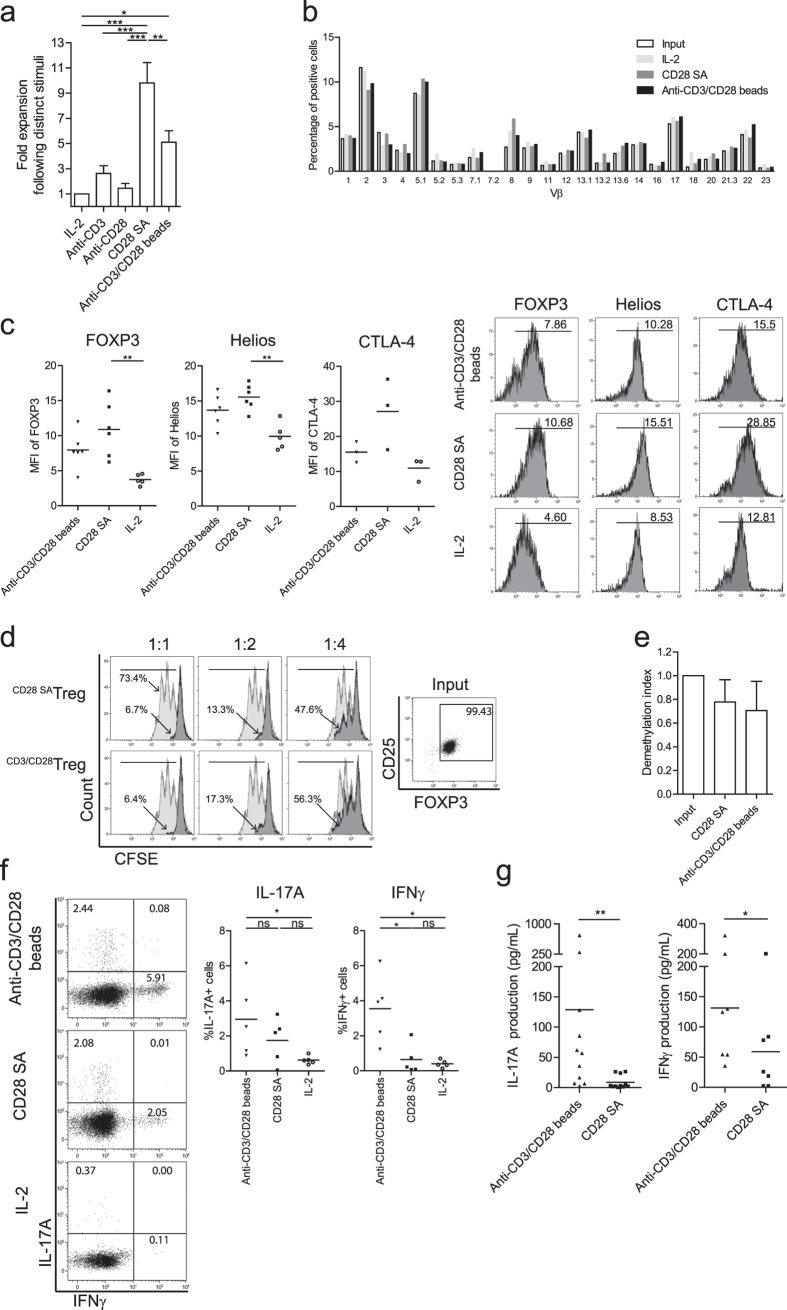
CD28-superagonist drives polyclonal Treg proliferation and enhances expression of FOXP3, Helios and CTLA-4. Flow cytometry of FACS-sorted human Treg that were stimulated with soluble CD28-superagonist(CD28 SA) or anti-CD3/CD28 mAb coated microbeads (anti-CD3/CD28 beads) in the presence of rhIL-2. As a control Treg cultured in the presence of rhIL-2 only were included. (**a**) Fold expansion of Treg during 7 days of culture. n = 6–9. (**b**) TcR Vβ repertoire analysis following the indicated stimuli (see legend). One experiment of two similar ones conducted with cells obtained from different donors is shown. (**c**) Intracellular expression of FOXP3, Helios and CTLA-4 by Treg after 7 days stimulation (X-axis). Numbers within the histogram show the median fluorescence intensity (MFI). n = 6. (**d**) CFSE-based co-culture suppression assays showing a representative experiment of two independent ones. The ratio of Treg:Tresp are indicated at the top. Numbers indicate the percentage of divided responder T cells. Grey line: stimulated Tresp; Black line: co-cultured with CD28-superagonist or anti-CD3/CD28 beads expanded Treg. (**e**) Demethylation status of *FOXP3* gene was analyzed using bisulfate sequencing. n = 3. (**f**) Intracellular staining of IL-17A and IFNγ at day 7 of the cultures. Numbers within the quadrant indicate the percentage of positive cells. Dot plots show a representative experiment of five individual ones conducted with cells obtained from different donors as shown in the cumulative data graphs; n = 5. (**g**) Measurement of IL-17A and IFNγ production in culture supernatants using luminex at day 7 of the cultures. n = 11 (for IL-17A) and n = 7 (for IFNγ). Bar in cumulative data indicated the mean value. Kruskal-Wallis followed by Dunns post-hoc test was used for statistical analysis (**a,c,e–g**). *P < 0.05; **P < 0.01; ns: not significant.

Using a CD28-agonistic mAb and exogenously added rhIL-2, we showed that stimulation of Treg with this CD28-agonist led to a Treg population with increased stability and good suppressor function, but lacked in full induction of proliferation. To improve this latter aspect, we stimulated Treg with the CD28-superagonist mAb ANC28.1, a strong inducer of human conventional T cell proliferation^26^. Indeed, as compared to CD28-agonist stimulation, CD28-superagonist stimulation led to a superior expansion of Treg, to a level significantly higher than that observed upon stimulation with anti-CD3/CD28 mAb coated microbeads (anti-CD3/CD28 beads, Fig. 3a, p < 0.05). Flow cytometry based TcR-Vβ analysis indicated a polyclonal expansion pattern in both the CD28-superagonist and anti-CD3/CD28 beads stimulated Treg (Fig. 3b). In contrast to anti-CD3/CD28 bead stimulated Treg, the expression levels of FOXP3 and Helios were significantly increased upon CD28-superagonist stimulation as compared to the IL-2 only culture condition (p < 0.01) (Fig. 3c). Likewise, a similar trend towards increased CTLA-4 expression was observed under CD28-superagonist stimulatory conditions. Moreover, the suppressor capacity (Fig. 3d) and TSDR demethylation (Fig. 3e) of Treg expanded by CD28-superagonist or anti-CD3/CD28 beads was similar. Notably, in line with CD28-agonist stimulation (as shown in Fig. 2c) and in contrast to anti-CD3/CD28 beads stimulated Treg, CD28-superagonist stimulated Treg showed a stable phenotype as evidenced by their low capacity to produce IFNγ (Fig. 3f and g). Although the percentage of IL-17A positive cells was not significantly reduced as compared to anti-CD3/CD28 beads stimulation, the production of IL-17A and IFNγ appeared in culture supernatants were significantly low (Fig. 3g, p = 0.0068 for IL-17A, p = 0.0313 for IFNγ). Taken together, this indicates that CD28-superagonist stimulation promotes proliferation of Treg while maintaining a stable Treg phenotype.

### CD28-superagonist stimulation drives both naïve and memory Treg proliferation

**Figure 4:**
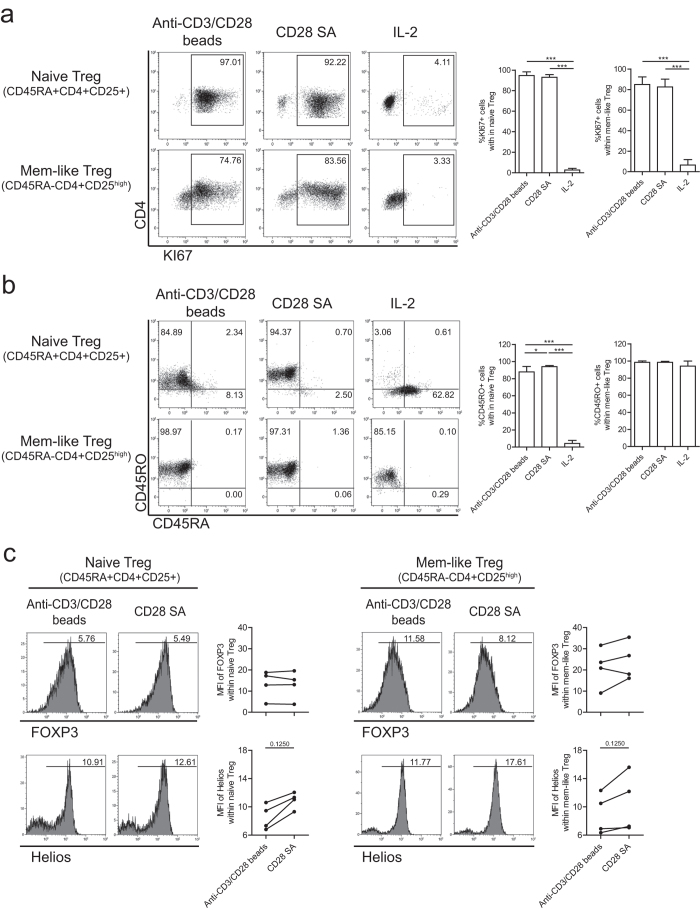
CD28-superagonist stimulation drives both naïve and memory Treg proliferation. Flow cytometry of FACS-sorted human naïve (CD4+CD45RA+CD25+) and memory (CD4+CD45RA-CD25^high^) Treg that were stimulated with CD28-superagonist(CD28 SA) or anti-CD3/CD28 mAb coated-microbeads in the presence of rhIL-2. As a control Treg cultured in the presence of rhIL-2 only were included. Dotplots show intracellular staining of KI67 (**a**), isoform switch of CD45RA and CD45RO (**b**), and intracellular expression of FOXP3 and Helios (**c**). Numbers within (**a**) and (**b**) indicate the percentage of positive cells, and numbers within the histogram (**c**) indicate the MFI values. A representative example of n = 4 individual experiments conducted with cells obtained from different donors is shown. Aggregate data are shown in the figures at right side of the flow cytometry plots. Kruskal-Wallis followed by Dunns post-hoc test (**a,b**) and Wilcoxon signed-rank test (**c**) were used for statistical analysis. *P < 0.05; ***P < 0.001.

It is debated whether naïve (CD4+CD45RA+CD25+) or rather memory-like (CD4+CD45RA-CD25^high^) Treg are the ideal candidate cell population to be used for Treg-based cell therapy^27^,^28^. Therefore, we separated human Treg into naïve and memory-like subsets based on the expression of CD45RA using FACS sorting (see Supplementary Fig. S1) and stimulated the cells with CD28-superagonist or anti-CD3/CD28 beads in the presence of rhIL-2 as described above. Comparable to anti-CD3/CD28 beads stimulation, CD28-superagonist stimulation led to proliferation of both naïve and memory-like Treg as indicated by the expression of cell division marker KI67 (Fig. 4a). Phenotypic analysis showed that CD28-superagonist, like anti-CD3/CD28 beads stimulation, induced an almost complete isoform switching from CD45RA to CD45RO in the naive Treg population (Fig. 4b). CD28 superagonist stimulation led to a trend towards increased Helios expression levels (MFI), a key stabilizing Treg transcription factor^29^, in both naïve and memory-like Treg subsets while CD28 SA and anti-CD3/CD28 bead stimulated Treg revealed similar FOXP3 expression levels (Fig. 4c). Together this implies that CD28-superagonist is of interest for the *ex vivo* expansion of both naive and memory-like Treg.

### The mTOR and PI3K pathways differentially regulate CD28-superagonist mediated Treg stability

**Figure 5:**
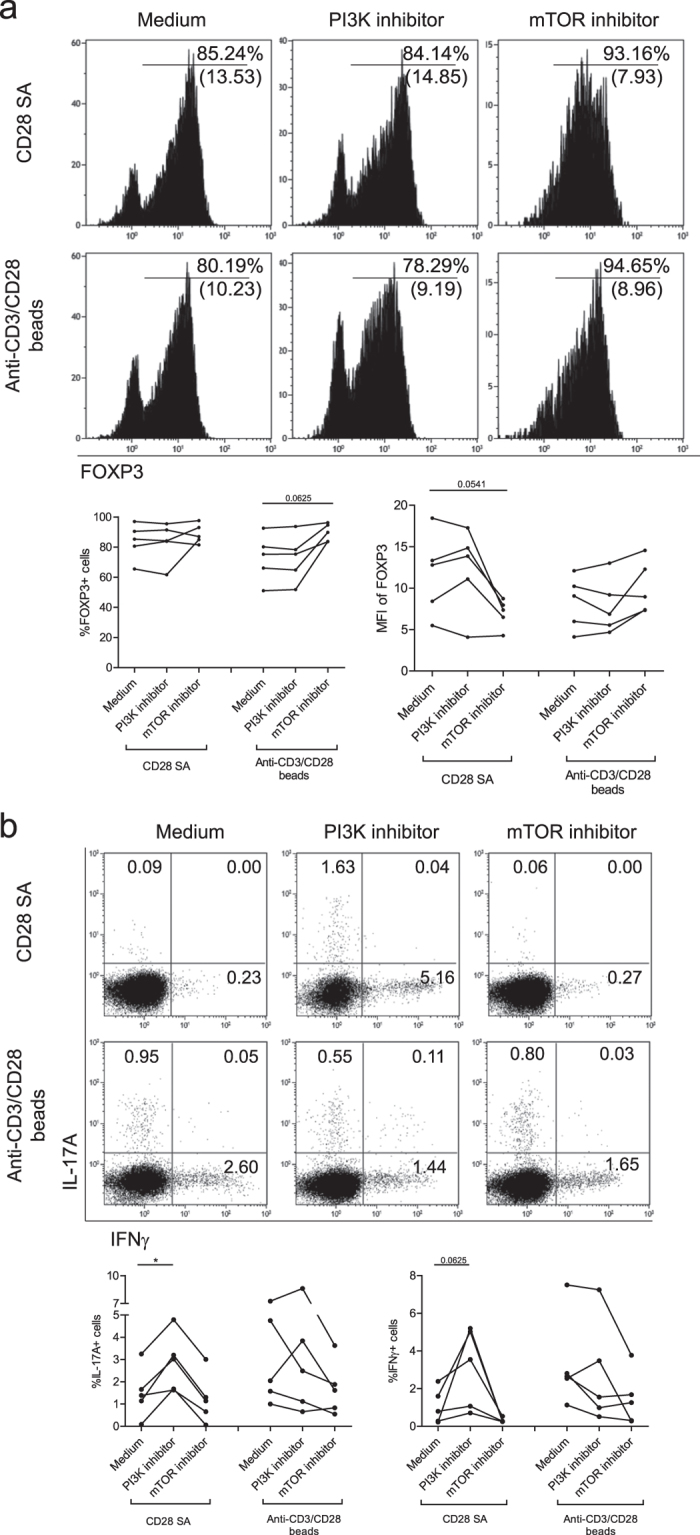
PI3K and mTOR differentially regulate CD28-superagonist mediated Treg stability. Flow cytometry of FACS-sorted human CD4+CD25^high^ Treg that were stimulated with CD28-superagonist(CD28 SA) or anti-CD3/CD28 mAb coated-microbeads and exogenously added rhIL-2. The cells were pretreated for 30 minutes with the PI3K inhibitor wortmannin, or mTOR inhibitor rapamycin as indicated on the top. (**a**) Intracellular FOXP3 expression and (**b**) intracellular IL-17A and IFNγ staining at day 7 of the cultures. Numbers within the histogram indicate the MFI of FOXP3 and the percentage of positive cells, respectively (**a**), and numbers within the quadrants indicate the percentage of cytokine producing cells. A representative example of n = 5 (**a,b**) individual experiments conducted with cells obtained from different donors is shown. Aggregate data are shown in the figures bellow the flow cytometry plots. Wilcoxon signed-rank test was used for the comparison between medium group and inhibitor-treated group. *P < 0.05.

CD28 stimulation is well known for its ability to activate the phosphoinositide-3 kinase (PI3K) pathway and its downstream targets such as mTOR^30^. To test if these signalling pathways are involved in CD28-superagonist mediated preservation of Treg, FACS sorted Treg were stimulated with the CD28-superagonist and exogenously added rhIL-2 in the absence or presence of a PI3K-inhibitor (wortmannin) or mTOR-inhibitor (rapamycin). In CD28-superagonist stimulated Treg the percentage of FOXP3 expressing cells was hardly influenced by PI3K or mTOR inhibition, but mTOR inhibition led to reduced FOXP3 expression levels per cell (p = 0.0541) (Fig. 5a). Regarding the cytokine producing potentials, PI3K inhibition led to a significant increase of IL-17A producing cells (Fig. 5b) and a similar trend was observed for IFNγ (Fig. 5b). mTOR-inhibition of CD28-superagonist stimulated Treg seemed to further inhibit the IFNγ producing capacity while hardly influencing the IL17A producing potential (Fig. 5b). In contrast to CD28-SA stimulated Treg and as reported previously^31^,^32^, in anti-CD3/CD28 stimulated Treg mTOR inhibition promotes FOXP3 expression (Fig. 5a). These findings suggest that the increase in FOXP3 expression and the reduced cytokine producing potential in CD28-SA stimulated Treg differentially depends on distal mTOR and proximal PI3K signaling of the PI3K-Akt-mTOR pathways respectively.

## Discussion

For safe and effective clinical Treg-based immunotherapy, the ability to select and expand stable and highly suppressive Treg from the peripheral derived Treg pool is a prerequisite. In the present study, we show that single CD28-stimulation of human peripheral Treg results in polyclonal expansion and preservation of a stable Treg phenotype and function as indicated by high level FOXP3/Helios expression, reduced expression of pro-inflammatory cytokines and potent suppressor function. Moreover, single CD28-stimulation of Treg, in contrast to anti-CD3 and anti-CD28 stimulation, results in reduced plasticity as indicated by the reduced potential to produce IL-17A and the inability to produce IFNγ. CD28-superagonist stimulation drives polyclonal expansion of peripheral Treg to a significantly higher level as compared to CD28-agonist and the standard anti-CD3/CD28 mAb-beads stimulation. As well, CD28-superagonist stimulation promotes expansion of both naïve and memory-like Treg. Finally, using pharmaceutical inhibitors of mTOR and PI3K signaling pathways we reveal that both signaling molecules play a differential mechanistic role in CD28-superagonist mediated preservation of Treg stability. Together, our results demonstrate that CD28-superagonist stimulation could serve to improve Treg *ex vivo* expansion protocols to obtain sufficient numbers of stable committed Treg.

CD28 is the best-characterized costimulatory molecule on T cells. CD28 stimulation is crucial for optimal naive T cell activation, cytokine production, proliferation, and survival^33^. With regard to Treg, CD28 stimulation plays an important role in the development of FOXP3+ cells in the thymus as CD28 or CD28-ligand deficient mice reveal reduced numbers of naturally occurring Treg and hence develop severe autoimmunity^18^,^19^. Recent new insights obtained in CD28-conditional knockout mice, where CD28 expression in FOXP3+ Treg is post thymically affected, supports the notion that CD28 also plays an important role in the proliferation and survival of Treg after thymic generation^20^. Indeed, this specific CD28 deficiency in Treg resulted autoimmune disease was prevented by supplementation with wild type CD28 expressing Treg^20^. This made us realize that CD28 signaling might also play an important role in Treg homeostasis. Therefore we here addressed the question as to whether single-CD28 stimulation might drive stable expansion of human Treg. In fact, we demonstrate that single-CD28 stimulation, in the absence of CD3 stimulation, as compared to anti-CD3/CD28 stimulated Treg, promotes superior Treg stability as indicated by increased FOXP3 expression and reduced production of pro-inflammatory cytokines.

Superagonistic CD28–specific mAbs are potent inducers of T cell proliferation both *in vitro* and *in vivo* in the absence of TCR engagement. Agonistic anti-rat CD28 mAb JJ316 supports large-scale expansion of rat CD4+CD25+ Treg cells in the absence of T-cell receptor stimulation^21^,^22^ *in vitro* and prevents EAE and adjuvant arthritis in rats^34^,^35^. These studies rationalized a phase I clinic trial of a humanized anti-human CD28-superagonist mAb, TGN1412, that lacked toxicity in non-human primates^36^. The trial was stopped due to rapid induction of multi-organ failure in six volunteers receiving the mAb that was likely caused by a cytokine storm. In our current work we have been studying ANC28.1mAb, a commercially available CD28-superagonist that possesses similar signaling signatures and functional properties as TGN1412^37^. In contrast to our findings, it has been postulated that high purity FACS-sorted human Treg did not respond to ANC28.1 stimulation after 3 days^26^. However, it is generally known that high purity sorted Treg require more time and supplementation with exogenous rhIL-2 to enter cell division. In fact, we here show that prolonged (7 days) stimulation of high purity sorted Treg by a CD28-superagonist in the presence of exogenously added rhIL-2 results in vigorous proliferation of both highly pure FOXP3+ naïve and memory-like Treg. Moreover, we found that all of the Treg input participated to proliferation after CD28-superagonist stimulation in the presence of rhIL-2, indicating that CD28-superagonist stimulation result in polyclonal expansion of Treg. Importantly, these expanded Treg revealed high and stable FOXP3 expression, limited pro-inflammatory cytokines producing potential and potent suppressor capacity. Although CD28-superagonist stimulation is not specific for Treg, we clearly demonstrate the great potential of CD28-superagonist in stable *ex vivo* expansion of human Treg. Interestingly, it was recently demonstrated that *in vitro* stimulation of human PBMC by low-dose CD28 superagonist (TGN1412) selectively activated Treg^24^. Recent work from Brunstein *et al*. provided further support for a role for CD28 mediated activation in Treg expansion by showing that artificial antigen presenting cells modified to express CD86, the natural ligand of CD28, resulted in enhanced Treg expansion as compared to stimulation with anti-CD3/CD28 beads^23^. Despite a more pronounced stable phenotype of CD28 SA expanded Treg as indicated by the lower IL-17A/IFNγ cytokine producing potential as compared to anti-CD3/CD28 expanded Treg, CD28 SA and anti-CD3/CD28 expanded Treg revealed similar *in vitro* suppressive potential (Fig.3). It has been demonstrated before that human cytokine producing Treg reveal similar suppressive capacity as Treg that do not produce cytokines^12^.

Both naïve (CD4+CD45RA+CD25+) and memory-like (CD4+CD45RA-CD25^high^) Treg populations have been characterized in human peripheral blood^38^. It was suggested that naïve Treg would be the preferred starting population for the homogenous expansion of stable Treg^27^. Here we show that CD28-superagonist activation drives proliferation of naïve as well as memory-like Treg and results in a trend towards increased expression levels of Helios on both Treg subsets. Therefore, CD28-superagonist stimulation is of interest for the *ex vivo* expansion of both naive and memory-like Treg subsets.

CD28 activation is known to enhance PI3K activity and its downstream targets Akt and mTOR^30^, and the PI3K-Akt-mTOR signaling network regulates FOXP3 expression^39^. In mice PI3K signaling plays a dualistic role on Treg induction and Treg maintenance^40^,^41^,^42^. We here demonstrate that PI3K and mTOR signaling play a differential role in stabilizing Treg following CD28-superagonist stimulation as PI3K-inhibition restored the IL17A and IFNγ producing potential whereas mTOR inhibition reduced FOXP3 expression levels. In contrast, in anti-CD3/CD28 stimulated Treg mTOR inhibition led to an increase in FOXP3 expressing cells and a reduced IFNγ producing potential, whereas PI3K inhibition hardly affected the IL17A and IFNγ producing potential. Here we show that mTOR plays a role in increased FOXP3 expression of CD28-expanded Treg, while mTOR, as has been shown in anti-CD3/CD28 stimulated Treg, may negatively regulate FOXP3 expression. The latter is in line with previous work showing that mTOR inhibition by rapamycin of *in vitro* stimulated human FACS isolated naturally occurring CD4+CD25^high^ Treg promotes selective outgrowth of FOXP3+ cells^15^,^43^. Apparently, multiple and different signaling pathways contribute to Treg stability in CD28-superagonist and CD3/CD28 beads expanded Treg. Together our findings indicate that PI3K and mTOR have a differential role in promoting the stability of CD28-expanded human peripheral Treg.

Summarizing, here we demonstrate that CD28-superagonist mediated *ex vivo* expansion of human Treg, which mechanistically depends on differential PI3K and mTOR-signalling, might be a promising new avenue for the expansion of stable polyclonal Treg intended for Treg-based immunotherapy in transplantation and autoimmunity.

## Methods

### Cell isolation

Peripheral blood mononuclear cells (PBMCs) were isolated using density gradient centrifugation (Lymphoprep, Nycomed Pharma AS, Oslo, Norway) from buffy coats of healthy blood donors, purchased from Sanquin Blood Supply Foundation, region South-East, Nijmegen, the Netherlands. Buffy coats were obtained upon written consent from the donor for scientific use according to the Dutch law. CD4+ cells were isolated by negative selection using RosetteSep^TM^ human CD4+ cell enrichment cocktail (Stemcell, Cologne, Germany). Thereafter, enriched CD4+ cells were labeled with anti-CD25-PeCy7 (M-A251; BD Biosciences, Breda, Netherlands) conjugated mAb. Thereafter, CD4+CD25^high^ Treg were sorted using a FACSAria cell-sorter (BD Bioscience). In case of naïve and memory Treg sorting, CD4+ cells were labeled with anti-CD25-PeCy7(M-A251) and anti-CD45RA-pacific blue (HI100, Biolegend, San Diego, CA, USA) mAbs; thereafter CD4+CD45RA+CD25+ naïve Treg and CD4+CD45RA-CD25^high^ memory Treg were sorted using a FACSAria cell-sorter. Cell purity was typically over 97%.

### Cell culture

Treg were stimulated with rhIL-2 (25 U/mL) alone as control, or together with either soluble CD28 agonist mAb (1 μg/mL, Clone L293, Cat# 348040; BD Bioscience), plate-bound CD3 mAb (5 μg/mL, Clone UCHT1, BD Bioscience), plate-bound CD3 (5 μg/mL) plus soluble CD28 mAb (1μg/mL), CD28-superagonist ANC28.1 (1 μg/mL, Clone ANC28.1/5D10, Cat# 177–820, preservative free; Ancell, Bayport, USA), or anti-CD3/anti-CD28 mAb-coated microbeads (Invitrogen by Life technologies, Bleiswijk, The Netherlands) used in a 1:2 bead-to-cell ratio. Titration of soluble CD28-agonist and CD28-superagonist antibodies at a concentration range of 1–10 μg/mL led to similar FOXP3 expression levels as determined by flow cytometry. Cells were cultured in 96-well round bottom plates in RPMI 1640 (Invitrogen) as described previously^44^. In selected experiments immunomodulating agents were analyzed; Treg were pretreated with wortmannin (5 μM), rapamycin (200 nM) (Sigma-Aldrich, St. Louis, USA)^15^, or vehicle control for 30 min at 37 °C. Thereafter, stimulators as indicated were added to the culture mixture.

### Flow cytometry, antibodies and CFSE labelling

Cells were phenotypically analyzed using multi-color flow cytometry (Beckman-Coulter, Brea, CA, USA). Antibodies used for flow cytometry include: anti-CD3-PeCy5 (UCHT1), anti-CD4-PC5.5 (13B8.2), anti-CD27-PC5.5 (1A4CD27) (all from Beckman-Coulter), anti-CD25-PeCy7 (M-A25, BD). Anti-FOXP3-eFluo660 (PCH101) or -eFluo450 (PCH101), anti-IL-17A (eBio64DEC17)-AlexFluo88 and anti-IFNγ(4S.B3)-Pcy7 (all from eBioscience, San Diego, USA), anti-Helios-AlexFluo647 (22F6, Biolegend), anti-CTLA4-PE (BNI3) and anti-active caspase 3-PE (C92–605) (both from BD) were used after Fix-Perm-treatment (eBiosciences). Appropriate isotype mAbs were used to define marker settings. Fixable viability dye eFluor^®^780 (eBioscience) was used in some experiments. Data were analyzed using Kaluza software (Beckman-Coulter).

To measure the intracellular cytokine producing potential, the cells were stimulated with PMA (12.5 ng/mL) plus ionomycin (500 ng/mL) in the presence of Brefeldin-A (5 μg/mL) for 4 hours before analysis. To analyze the TCR-Vβ repertoire by flow cytometry we used a TCR-Vβ kit (Betamark, Beckman-Coulter) according to the manufacturer’s instructions. To monitor cell division by flow cytometry, cells were labeled with 0.5 μM CFSE (Invitrogen) and analyzed by flow cytometry as described previously^45^.

### Cytokine detection assay

IL-17A and IFNγ were determined in the culture supernatants using Luminex cytokine assays (Invitrogen), according to the manufacturer’s instructions. The lower level of detection of IFNγ was < 5 pg/mL and that of IL-17A was < 10 pg/mL.

### Co-culture suppression assays

The suppressor capacity of expanded-Treg was studied in co-culture suppression assays. Treg were cultured for 7 days under the conditions described above. Thereafter, viable Treg were collected, washed and added at different ratios to CFSE-labeled CD4+CD25- responder cells (Tresp) together with anti-CD3/anti-CD28 mAb-coated beads at a bead-to-cell ratio of 1:5 for 3 days. Proliferation of Tresp was determined by analyzing CFSE dilution as described previously^45^.

### FOXP3 gene methylation

The methylation status of *FOXP3* gene was analyzed by bisulphate sequencing, as described previously^46^. In brief, geneGenomic DNA was isolated from Treg using the QIAamp DNABloodMini kit (Qiagen, Venlo, Netherlands), Bisulfite converted and amplified using bisulfite-specific polymerase chain reaction (PCR) (forward 59 TGGATATTTGGTTAGAGTTAA GAAT 39 and reverse 59 ACCTAACACTCTCAAAACTTCAAAC 39). The purified PCR product was sequenced on an ABI 3130 Genetic Analyzer (Applied Biosystems by Lifetechnologies), and analyzed using Sequencing Analysis version 5.4 software.

Cumulative methylation status data is presented as demethylation index, calculated by percentage of demethylation after Treg culture/percentage of demethylation of the input Treg population. Percentage of demethylation of FACS-sorted input Treg is typically > 95%^46^.

### Statistics

Data derived from two or more than two groups with non-normal distributions were compared using Wilcoxon matched-pairs test or Kruskal-Wallis test, respectively. The multiple comparisons were adjusted with a Dunns post-hoc test to compare groups involved. P < 0.05 was considered statistically significant. These statistical tests were performed in GraphPad Prism 5.0 for Windows (GraphPad Software Inc., La Jolla, USA).

## Additional Information

**How to cite this article:** He, X. *et al*. Single CD28 stimulation induces stable and polyclonal expansion of human regulatory T cells. *Sci. Rep.* **7**, 43003; doi: 10.1038/srep43003 (2017).

**Publisher's note:** Springer Nature remains neutral with regard to jurisdictional claims in published maps and institutional affiliations.

## Supplementary Material

Supplementary Figure S1
