# Supplementary material for: Single CD28 stimulation induces stable and polyclonal expansion of human regulatory T cells
Source: Sci Rep. 2017 Feb 22;7:43003. doi: 10.1038/srep43003 (PMC5320448; doi:10.1038/srep43003)
Supplement: Supplementary Figure S1 [file srep43003-s1.pdf]

# Single CD28 stimulation induces stable and polyclonal expansion of human regulatory T cells

Xuehui He[1,2], Ruben L. Smeets[3], Esther van Rijssen[1], Annemieke M.H. Boots[4], Irma Joosten[1] and Hans J.P.M. Koenen[1,*]

[1]Laboratory of Medical Immunology, Department of Laboratory Medicine, Radboud university medical center, Nijmegen, The Netherlands; [2]College of Computer Science, Qinghai Normal University, Xining, Qinghai, China; [3]Laboratory of Clinical Chemistry, Department of Laboratory Medicine, Radboud university medical center, Nijmegen, The Netherlands; [4]Department of Rheumatology and Clinical Immunology, University of Groningen, University Medical Center Groningen, Groningen, The Netherlands.

***Contact information for correspondence:***

* Dr. Hans J.P.M. Koenen
Department of Laboratory Medicine - Medical Immunology (route 469)
Radboud university medical center
Geert Grooteplein zuid 10
Postbus 9101, 6500 HB Nijmegen
The Netherlands
Email: hans.koenen@radboudumc.nl

# Supplemental Figure S1

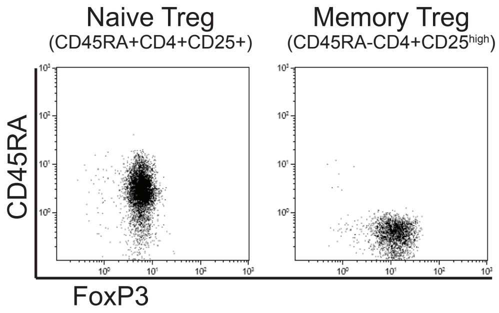


**Supplemental Figure S1: FOXP3 expression on freshly sorted human naïve and memory Treg.** Purified human CD4+ T cells were stained with CD45RA and CD25. Thereafter, CD45RA+CD25+ naïve Treg and CD45RA-CD25high memory Treg were FACS sorted. Subsequently, intracellular expression of FOXP3 was analysed in the sorted naïve and memory Treg by flow cytometry. Dotplots show a representative experiments of two individual experiments conducted with cells obtained from two different donors.
